# Reducing VEGFB accelerates NAFLD and insulin resistance in mice via inhibiting AMPK signaling pathway

**DOI:** 10.1186/s12967-022-03540-2

**Published:** 2022-07-30

**Authors:** Rongrong Li, Yuqi Li, Xueling Yang, Yaorui Hu, Haining Yu, Yana Li

**Affiliations:** 1 Department of Pathophysiology, School of Basic Medicine, Binzhou Medical University, Yantai, 264000 Shandong China; 2 Shandong Technology Innovation Center of Molecular Targeting and Intelligent Diagnosis and Treatment, Binzhou Medical University, Yantai, 264000 Shandong China; 3 Department of Anatomy, School of Basic Medicine, Binzhou Medical University, Yantai, 264000 Shandong China; 4 Stomatology Department, Stomatological College, Binzhou Medical University, Yantai, 264000 Shandong China

**Keywords:** VEGFB, NAFLD, AMPK/ACC, AMPK/SREBP1, Lipid metabolism

## Abstract

**Objective:**

Vascular endothelial growth factor B (VEGFB) was regarded to improve lipid metabolism and reduce obesity-related hyperlipidemia. Whether VEGFB participates in lipid metabolism in nonalcoholic fatty liver disease (NAFLD) has not been clear yet. This study investigated the involvement of VEGFB in lipid metabolism and insulin resistance via the AMPK signaling pathway in NAFLD.

**Methods:**

We constructed the animal and cell model of NAFLD after VEGFB gene knockout to detect liver damage and metabolism in NAFLD. Bioinformatics analysis of VEGFB and the AMPK signaling pathway relative genes to verify the differential proteins. And mRNA levels of NAFLD fatty acid metabolism-related genes were detected.

**Results:**

After the systemic VEGFB knockout mice were fed with high fat, the body fat, serum lipoprotein, NAFLD score, and insulin resistance were increased. Animal and cell experiments showed that the expression levels of phosphorylated proteins of CaMKK2 and AMPK decreased, the expression of proteins related to AMPK/ACC/CPT1 signaling pathway decreased, and the target genes CPT1α and Lcad decreased accordingly, reducing fatty acid oxidation in hepatocyte mitochondria; The expression of AMPK/SREBP1/Scd1 signaling pathway relative proteins increased, ACC1 and FAS increased correspondingly, which increased lipid synthesis in the endoplasmic reticulum.

**Conclusion:**

VEGFB can participate in lipid metabolism and insulin resistance of NAFLD through the AMPK signaling pathway.

**Supplementary Information:**

The online version contains supplementary material available at 10.1186/s12967-022-03540-2.

## Background

NAFLD is a clinicopathological syndrome characterized by additional lipid deposition in hepatocytes caused by reasons except for alcohol and other certain liver injury factors, which will lead to a series of liver injuries, including simple fatty liver (SFL), nonalcoholic steatohepatitis (NASH) and liver cirrhosis [1]. NAFLD has become a major public health problem of global concern. It is predicted that from 2016 to 2030, the morbidity of NAFLD will increase with the growth of morbidity of diabetes and obesity. By 2030, more than 1/3 of the total population will be diagnosed with fatty liver disease, and a considerable number of them will develop into fatty hepatitis [2, 3].

NAFLD is a continuous liver injury, from simple hepatic steatosis to liver fibrosis, cirrhosis, and liver failure. The most distinct feature is the disorder of lipid metabolism [1, 4]. The new vision of NAFLD research also increasingly focuses on the organelles closely related to lipid metabolism in hepatocytes [5]. For example, the accumulation of LD in hepatocytes is a significant feature of NAFLD. Lipids can change the intracellular Ca^2+^ homeostasis, promote the accumulation of LDs, and promote theβoxidation of fatty acids on mitochondria reduced [6, 7].

In recent years, VEGFB participation in lipid metabolism has attracted many scholars’ attention. VEGFB, as a homolog of VEGF-A, was founded in 1996 and was produced in the form of a secretory homodimer [8]. VEGFB has particular and unusual functional characteristics and does not show significant activity under normal conditions. In 2010, Hagberg et al. first reported in Nature that VEGFB can control endothelial cells taking fatty acids by regulating fatty acid transporters [9]. In 2016, Robciuc et al. also found that the VEGFB gene was transduced into obese mice with HFD, which can inhibit obesity-related inflammation and improve lipid metabolism [10]. Studies have confirmed that VEGFB can inhibit HFD-induced weight gain to reduce obesity and ameliorate insulin resistance [11].

The activation of the AMPK signaling pathway improves insulin resistance and lipid accumulation in HepG2 cells [12]. Studies have shown that the mechanism of AMPK reducing liver lipids is to inhibit newborn fat synthesis and promote fatty acid oxidation [13]. Sterol regulatory element binding protein-1 (SREBP1), as a key protein downstream the AMP-activated protein kinase (AMPK), participates in the process of fatty acids synthesis. In addition, acetyl coenzyme A carboxylase (ACC) and fatty acid synthase (FAS) are also involved in it [14].

The AMPK/ACC/CPT1 signaling pathway has positive significance in reducing lipid accumulation and improving lipid metabolism [15]. AMPK inhibits target protein ACC by phosphorylation and enhances carnitine palmitoyltransferase 1 (CPT1) activity to reduce fatty acid synthesis. Besides, SREBP1 is the main regulator of FAS and other adipogenic proteins. AMPK regulates fatty acids synthesis of the transcriptional target protein stearoyl CoA Desaturase-1 (Scd1) [16, 17]. The overexpression of the AMPK/ACC/SREBP1 pathway effectively inhibited hepatic steatosis, improved lipid and glucose metabolism, and ameliorated damaged liver function β oxidation [18]. The AMPK/ACC/CPT1 pathway and the AMPK/SREBP1/Scd1 pathway may be potential targets for the prevention of hepatic lipid deposition and the treatment of NAFLD. By constructing NAFLD mice and cell models, we observed the pathological changes of liver injury, and detected the key proteins in the AMPK/ACC/CPT1 and the AMPK/SREBP1/Scd1 pathways. Our results showed that VEGFB gene suppression can reduce the level of AMPK phosphorylation through CaMKKβ, inhibit ACC/CPT1 pathway and affect fatty acid oxidation, meanwhile, it could promote the SREBP1/Scd1 pathway and lead to lipogenesis and accelerate the development of NAFLD.

## Methods

### Crispr/cas 9 technology

**Fig. 1:**
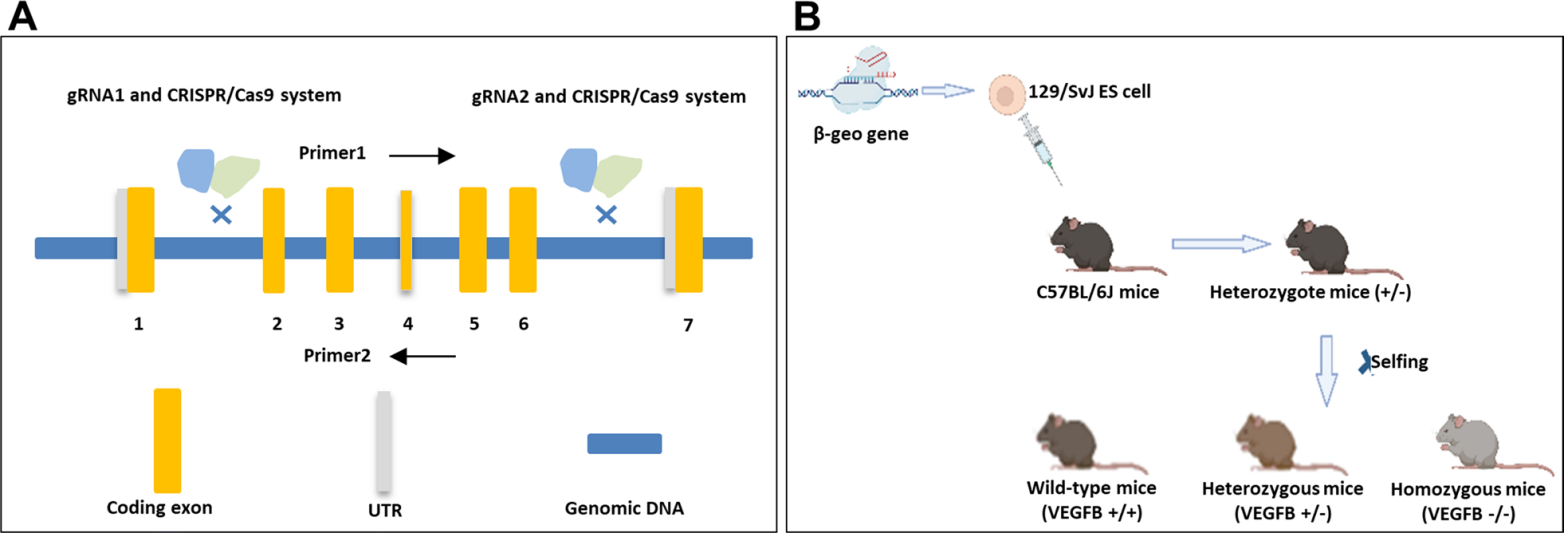
The construction of VEGFB gene knockout mice. **A** The structure of VEGFB gene. **B** Process of constructing systemic of VEGFB gene knockout mice by Crispr/Cas9

The VEGFB gene knockout mouse model was constructed by Guangzhou Saiye biotechnology company using the Crispr/cas 9 system. VEGFB gene ID is 22340, locates on chromosome 19, and NM_011697.3 transcripts are selected as reference transcripts. This transcript has 7 exons, ATG is on exon 1 and TAG is on exon 6. There are 90% of the protein-coding region of exon 2–6 of the VEGFB gene was knocked out in a large fragment which was about 2000 bp during the experiment. The first 30 amino acids of exon1 in the VEGFB gene were reserved. The cas9 plasmid was constructed, which could express sgRNA (Fig. 1A). The fertilized eggs were microinjected with sgRNA and cas9 mRNA into the oviduct of C57BL/6 mice, and the 0 generation VEGFB positive heterozygous mice (VEGFB^±^) (F0 mice) were obtained. After sexual maturation, F0 mice were mated with C57BL/6 mice, and F1 hybrid knockout mice were identified. Homozygous-type knockout mice (VEGFB^−/−^), heterozygous knockout mice and wild mice (VEGFB^+/+^) were obtained from F1 generation heterozygous knockout mice by selfing (Fig. 1B). PCR and Elisa verified the level of VEGFB knockout.

Only 1094 bp bands were shown in homozygous-type mice (VEGFB^−/−^) and 616 bp bands were shown in wild-type mice (VEGFB^+/+^).

### Animal

All animal experiments were approved by the animal ethics committee of Binzhou Medical University (IACUC Protocol Number: 2022-210), and the experimental animals maintained 12 h light–dark cycles. We selected 4-week-old, 16-18 g, C57BL/6 healthy male mice for the experiment, and used high-fat diet (20% kcal% protein, 20% kcal% carbohydrate and 60% kcal% fat), to construct the NAFLD mice model. During the experiment, no mice died of gene knockout, high-fat feeding, liver injury and other diseases. Normal C57BL/6 mice and VEGFB^−/−^ mice were grouped into three: SD group (Rodent Diet with 10% kcal% fat, n = 8), HFD group (Rodent Diet with 60% kcal% fat, n = 8) and HFD-VEGFB group (VEGFB^−/−^ mice with HFD containing energy fat of 60%, n = 8). We detected the body weight of mice during 16 weeks of HFD. On the last day of the study, the contents of fat, lean and liquid in mice were measured by body fat meter. The mice were killed after isoflurane anesthesia. The whole blood was obtained rapidly from the eyeball. The serum can be extracted for 200–300 μl after centrifugation at 2000 r/min for 10 min at 4 ℃ for Elisa. And the liver and adipose tissue of mice were collected for weighing and quickly frozen to store at – 80 ℃. Meanwhile, some liver tissue and adipose tissue were preserved in paraffin-embedded sections for histological analysis (Table 1).

**Table 1:** The ingredients of rodent high fat diet

Ingredients	gm	kcal
Casein, 30 Mesh	200	800
l-Cystine	3	12
Corn Starch	0	0
Maltodextrin 10	125	500
Sucrose	68.5	275.2
Cellulose, BW200	50	0
Soybean oil	25	225
Lard	245	2205
Mineral mix S10026	10	0
DiCalcium Phosphate	13	0
Calcium carbonate	5.5	0
Potassium Citrate,1 H2O	16.5	0
Vitamin mix V10001	10	40
Choline Bitartrate	2	0
FD&C blue dye #1	0.05	0
Total	773.85	4057

Ingredients are indicated in g for the preparation of 1 kg of each diet

### Cell culture and treatment

HepG2 cells were cultured in DMEM containing 10% FBS and 1% penicillin/streptomycin (P/S) in a cell incubator at 37 °C and 5% CO_2_ concentration. HepG2 cells were seeded in 6 well-culture plates with a density of 3 × 10^5^ cells/well. The NAFLD cell model was completed by 400 μM palmitic acid (PA) for 24 h to construct and grouped into two. The suspension was collected after centrifuge to detect TC and TG content for evaluation of constructing the cell model. HepG2 cells were seeded in 96-well culture plates with the density of 4 × 10^3^ cells/well and then incubated with 0 μM, 400 μM, 800 μM, 1000 μM PA solution, and 50 g/L BSA as a negative control for 24 h to detect the absorbance at 490 nm by a Synergr2 multifunctional microplate reader.

### SiRNA transfection

The cells were divided into two groups, SI group and NC group. In SI group, SiRNA targets to VEGFB were transfected into HepG2 cells with NAFLD using jetPRIME (Lot#0000000010, Polyplus, IIIkirch, France) and jetBuffer (Lot# B210723, Polyplus, IIIkirch, France). In NC group, we constructed a negative control with a nontargeting siRNA. Cells were replaced medium after 4 h transfection and continued to incubate for 48 h. RNA and protein were extracted to examine the transfection was completed.

### Body fat rate and organ ratio

At room temperature of 20–25 ℃, 20 weeks old mice were weighed and put into the body fat meter (E1452120, Bruker BioSpin GmbH, Rheinstetten, Germany) according to different groups. The contents of fat, lean and liquid in mice were detected. Then the mice were euthanized. The weight of white fat in the epididymis, white fat under inguinal skin and liver were weighed, and the organ ratio was calculated. Obesity index = white adipose tissue (white fat in epididymis, white fat under inguinal skin) divided by body mass × 100.

### Serological indicators

The mice were killed by breaking the neck, and the serum was taken from the eyeball blood after centrifuge. TG (Cat#A110-1-1), TCHO (Cat#A111-1-1), LDL (Cat#113-1-1), HDL (Cat#112-1-1), AST (Cat#C009-2-1), and ALT (Cat#C010-2-1) were detected according to the manufacturer's instructions (All from Nanjing Jiancheng Bioengineering Institute, China). According to the instructions of Shanghai enzyme-linked biological mouse insulin KIT (Cat #ml001983, Shanghai, China), the absorbance value was examined at 450 nm.

### Lipoprotein indicators in liver tissues and cells

The liver tissue of mice was added 9 times the volume of absolute ethanol according to the weight (g) and volume (ml) ratio of 1:9. After mechanical homogenization in the ice water bath, the supernatant was centrifuged at 2500 r/min for 10 min. HepG2 was digested by trypsin and centrifuged at 1000 r/min for 10 min to take the supernatant. The contents of TG and TCHO in liver tissues and cells were determined by Elisa according to the instructions of the manufacturer’s instructions.

### Morphological detection and NAFLD score

Mouse liver tissue was fixed with 4% neutral paraformaldehyde, and adipose tissue was fixed with adipose tissue fixing solution (Cat#g1119, sevicebio, Wuhan, China) and embedded in conventional paraffin. Sections were stained with HE and Masson trichrome fiber (Cat#1340, Solarbio, Beijing, China), observed and photographed under the microscope. Under the vision of 400X adipose tissue and 1000 × slice of liver tissue, the size and area of droplets and the area of steatosis of hepatocytes were counted by Image Proplus. According to the NAFLD scoring standard from Liang et al. the NAFLD scores of mice in each group were calculated by counting four parameters: macrovesicular steatosis, microtubular steatosis, hepatocyte hypertrophy, and inflammatory cell aggregation [19] (Table 2).

**Table 2:** The scoring system for NAFLD

Histological feature	Score			
	0	1	2	3
Macrovesicular steatosis	< 5%	5–33%	33–66%	> 66%
Microvesicular steatosis	< 5%	5–33%	33–66%	> 66%
Hypertrophy	< 5%	5–33%	33–66%	> 66%
Inflammation				
Number of inflammatory foci/field	< 0.5	0.5–1.0	1.0–2.0	> 2.0

### ITT and GTT

After fasting for 12 h overnight, mice were fed with 2 mg/kg glucose by gavage at 0, 15, 30, 60, 90, and 120 min, blood glucose was detected by a blood glucose meter at the tail, GTT curve was drawn, and the area under the curve was calculated. During ITT, the mice were injected intraperitoneally with insulin (0.5 U/kg) after fasting for 6 h. At 0, 15, 30, 60, 90, and 120 min after injection, the blood glucose was measured and the ITT curve was drawn.

### HOMA-IR and QUICKI

The insulin resistance indicators and insulin sensitivity indicators were calculated. HOMA-IR = fasting blood glucose (FPG) × Fasting insulin (FINS)/22.5; Insulin sensitivity indicators (QUICKI) = 1/(log FPG + log FINS).

### Bioinformatics analysis

Based on the genetwork website (http://genetwork.org), we analyzed the liver mRNA of SD and HFD mice in BXD family mice, screened the Top 2000 genes related to VEGFB and AMPK respectively, and screened their co-expressed gene network through the website. The WEB-based gene set analysis tool kit (WebGestalt) is used for gene set enrichment analysis.

### Western blot

Tissue and cell proteins were extracted with a mixture containing 1% protease inhibitor (Cat#A8260, Solarbio, Beijing, China) and RIPA lysate (Cat#R0010, Solarbio, Beijing, China). The protein was denatured by heating in a metal bath at 99 ℃ for 10 min. After calculating the volume with the same mass of 20 μg, the protein was loaded, and the protein was separated. The membrane was sealed with 5% milk for 1 h. p-AMPK (Cat#2535S, 1:1000, CST, USA), AMPK (Cat#2532S, 1:1000, CST, USA), CaMKKβ (Cat#DF4793, 1:1000, Affinity, USA), p-ACC (Cat#11818, 1:1000, CST, USA), ACC (Cat#3676, 1:1000, CST, USA), CPT1 (Cat#ab234111, 1:1000, Abcam, UK), SREBP1 (Cat#ab28481, 1:1000, Abcam, UK), Scd1 (Cat#ab236868, 1:1000, Abcam, UK). β-actin (Cat#3700, 1:1000, CST, USA) was incubated with membrane-binding protein overnight. The membrane was incubated with antibodies at room temperature for 1 h. The bands were analyzed in an enhanced chemiluminescence system (Clinx, Shanghai, China). The intensity of the band was measured by Image J (1.50i, National Institutes of Health, USA).

### Q-PCR

Total RNA in cells and tissue homogenate was extracted with Trizol reagent (Cat#15596-018, Invitrogen, CA, USA). Genomic DNA was removed with DNA wiper mix, and then the RNA was reversed into cDNA with Hiscript III super mix (Cat#R323, Vazyme, Nanjing, China). 2X SYBR qPCR Master Mix (Cat#Q711, Vazyme, Nanjing, China) mix was used to amplify the fluorescence quantitative PCR instrument (Thermofisher, Beijing, China) to analyze the expression of the target gene. The primer sequence is shown in the attached table (Table 3).

**Table 3:** Sequences of primer pairs used for real-time PCR

Gene	Primer	5′–3′ sequence
VEGFB	F R	AGCCACCAGAAGAAAGTGGT GCTGGGCACTAGTTGTTTGA
CPT1α	F R	CTCCGCCTGAGCCATGAAG CACCATGATGATGCCATTCT
Lcad	F R	TCACCAACCGTGAAGCTCGA CCAAAAAGAGGCTAATGCCATG
ACC1	F R	CGCTCAGGTCACCAAAAAGAAT GTCCCGGCCACATAACTGAT
FAS	F R	CTGAGATCCCAGCACTTCTTGA GCCTCCGAAGCCAAATGAGTGA
β-actin	F R	CATCCGTAAAGACCTCTATGCCAAC ATGGAGCCACCGATCCACA

*F* forward; *R* reverse

### Statistical analysis

Data are presented as the means + standard deviation (SD) and were analyzed using the Least Significant Difference (LSD) test; cut-off p < 0.05. Independent sample t-test and analysis of variance (ANOVA) were performed using the SPSS 20.0 software (SPSS Inc. Chicago, IL, USA).

## Results

### VEGFB gene suppression accelerates the lipid deposition in the whole body of mice and HepG2 cell

**Fig. 2:**
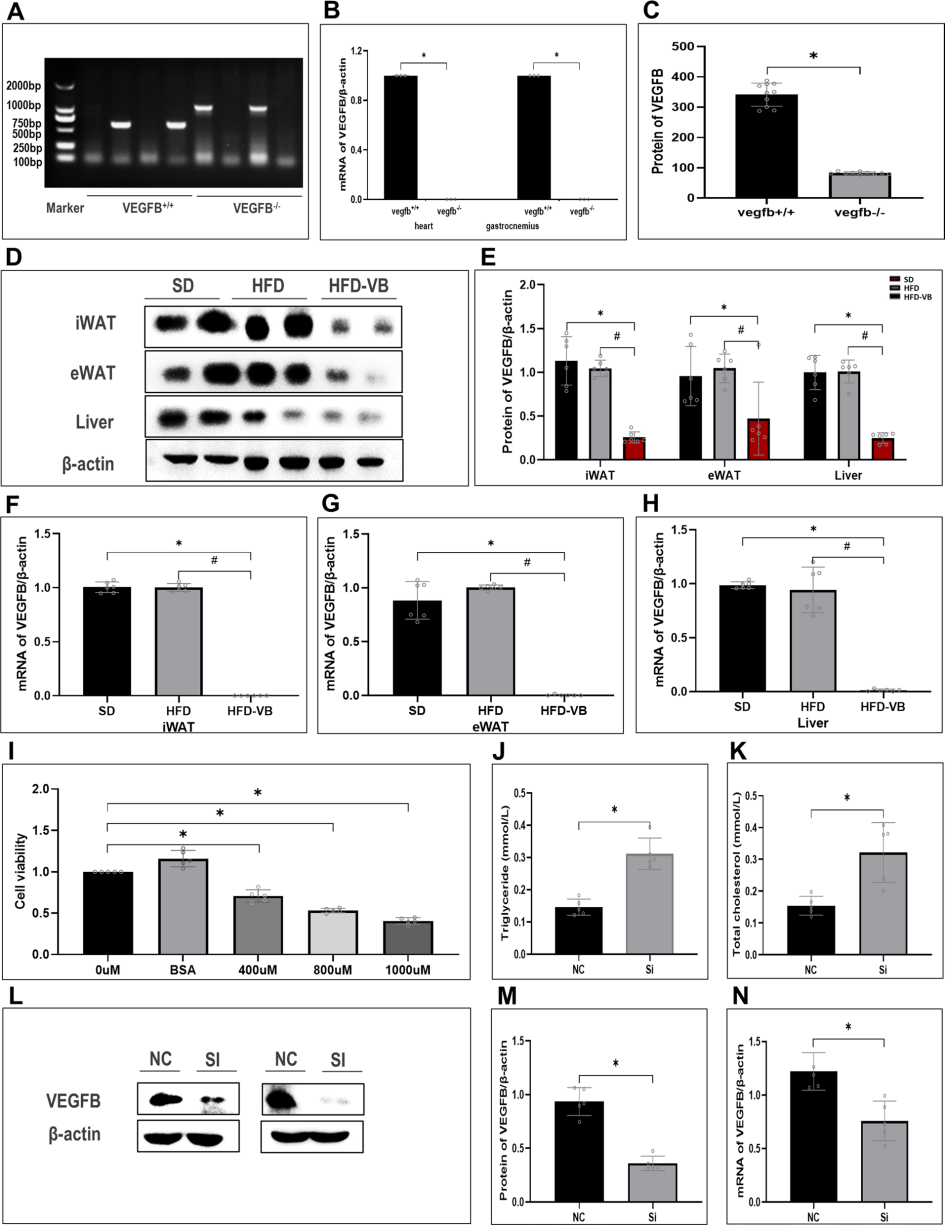
Appraisal of VEGFB gene knockout mice and NAFLD cell model with low expression of VEGFB. **A** VEGFB gene identification in wild-type and homozygous-type mice. **B** Relative mRNA expression level of VEGFB gene in heart and muscle tissue. **C** The content of VEGFB was detected by Elisa in the plasma of VEGFB systemic knockout wild-type and homozygous-type mice. **D** iWAT、eWAT and liver tissue of VEGFB gene knockout mice were analyzed by Western Blot. **E** VEGFB Protein expression level in iWAT、eWAT and liver tissue of VEGFB gene knockout mice. **F**–**H** Analyses of relative mRNA level of iWAT, eWAT and liver tissue in 3 groups of mice by RT-PCR. **I** HepG2 cell viability was induced by different concentrations of PA. **J**, **K** Levels of TG and TCHO in HepG2 induced by PA. **L** Western blot test of VEGFB in HepG2 cells after siRNA transfection. **M** Expression level of VEGFB protein in NAFLD cell model. **N** Relative mRNA expression level of VEGFB gene in NAFLD cell model. N = 6 cases per group. **A**–**C** * indicates vs. VEGFB^+/+^. **D**–**H** * indicates vs. SD, p < 0.05; # indicates vs. HFD, p < 0.05. **I**–**N** * indicates vs. NC, p < 0.05

We constructed a systemic VEGFB knockout mice model by Crispr/Cas9 technology (Fig. 2A). VEGFB was highly expressed in the heart and skeletal muscle of wild-type mice, and only slightly expressed in homozygous-type mice (Fig. 2B). The content of VEGFB in plasma of mice was detected by ELISA. The results showed that VEGFB was only slightly expressed in VEGFB^+/+^ mice, which was significantly lower than that of wild-type mice, indicating that VEGFB gene was successfully knocked out (Fig. 2C). The expressions of VEGFB in inguinal white fat (iWAT), epididymis white fat (eWAT) and liver of VEGFB knockout mice were markedly declined (Fig. 2D–H). We induced HepG2 with PA solution and constructed a NAFLD cell model. MTT found that the cell viability descended gradually at 400 µM, 800 µM and 1000 µM concentrations of PA solution (Fig. 2I). After HepG2 was induced by 1000 µM PA solution for 48 h, the contents of triglyceride (TG) and total cholesterol (T-CHO) increased markedly, indicating the construction of the NAFLD cell model was completed (Fig. 2J, K). And then, the results were examined at the protein and genetic levels after VEGFB was suppressed by siRNA transfection (Fig. 2L–N).

**Fig. 3:**
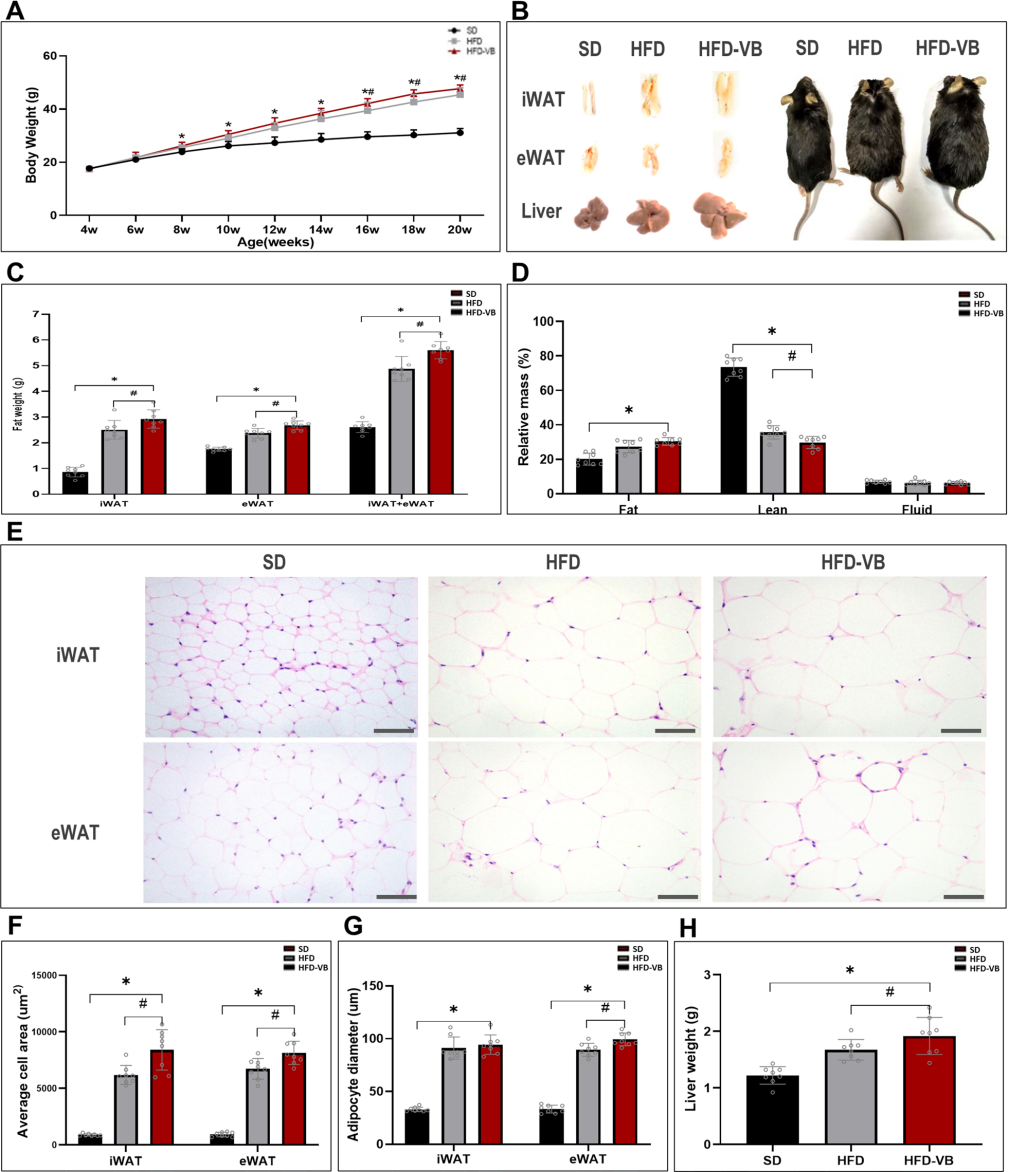
VEGFB gene knockout accelerated fat accumulation induced by HFD. **A** Monitoring of growth curve of mice aged 4–20 weeks. **B** Morphological pictures of iWAT, eWAT, liver and mice aged 20 weeks. **C** The relative weight of fat in 20-week-old mice. **D** The relative mass ratio of systemic fat, lean and liquid in 20-week-old mice. **E** HE staining pictures of iWAT and eWAT under a 400X light microscope. **F**, **G** Average area and average diameter of fat cells in iWAT and eWAT. **H** Weight of liver. N = 8 cases per group. * indicates vs. SD, p < 0.05; ^#^ indicates vs. HFD, p < 0.05

From the 4th week, we detected the effect of HFD on lipid accumulation in systemic VEGFB knockout mice. The results showed that from the 8th week of HFD, the weight of VEGFB knockout mice was markedly higher than that of SD mice. Compared with the HFD group, the weight difference of VEGFB knockout mice began to appear from the 16th week (Fig. 3A). VEGFB knockout mice have thickened inguinal white adipose and increased liver volume, and their body shape is more obese than that of SD and HFD mice (Fig. 3B). The weight of iWAT and eWAT of mice in HFD-VB group was significantly higher than that of mice in HFD group (Fig. 3C). We analyzed the body fat rate of living mice by a body fat analyzer and found that the content of adipose tissue was increased and muscle tissue was descended in VEGFB knockout mice after high-fat feeding, which was markedly different from that of SD and HFD mice (Fig. 3D). The area and diameter of adipocytes in inguinal white adipose tissue (iWAT) and epididymis white adipose tissue (eWAT) of VEGFB knockout mice were markedly increased (Fig. 3E–G). After high-fat feeding, the liver weight of VEGFB knockout mice increased markedly at the 20th week (Fig. 3H), and the body weight was also markedly higher than that of SD and HFD mice (Fig. 3A).

**Fig. 4:**
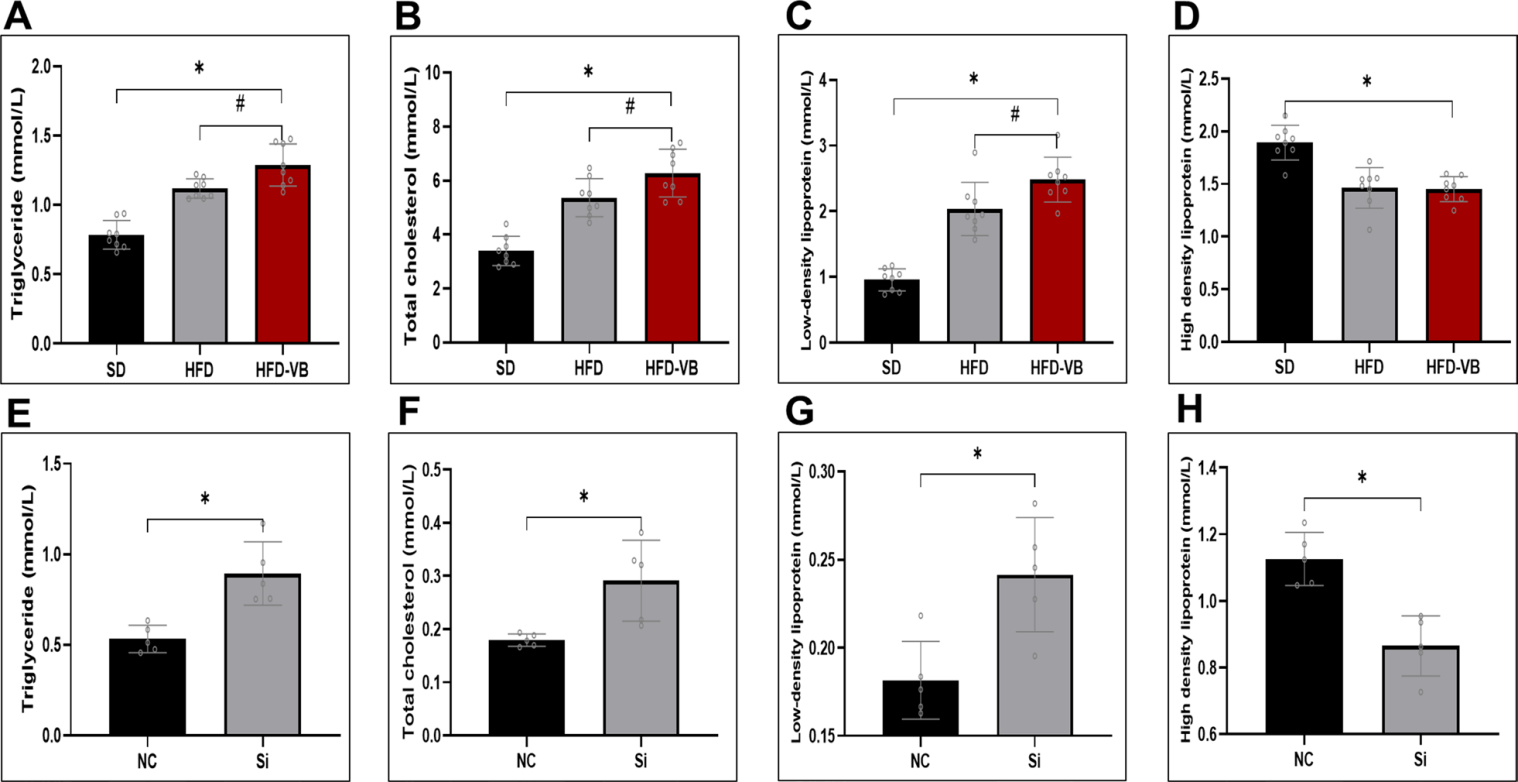
VEGFB gene suppression induced disorder of lipid metabolism in NAFLD mice. **A**–**D** Changes of TG、TC、LDL and HDL of plasma and serum in 20-week-old mice. **E**–**H** Changes of TG、TC、LDL and HDL in HepG2 cells induced by PA solution. N = 8 cases per group. **A-D** * indicates vs. SD, p < 0.05; ^#^ indicates vs. HFD, p < 0.05. **E**–**H** * indicates vs. NC, p < 0.05

To further confirm VEGFB on lipid metabolism, TG, T-CHO, low-density lipoprotein (LDL) and high-density lipoprotein (HDL) were detected in mouse serum and HepG2 cells respectively. The results revealed that the serum TG, TCHO and LDL of VEGFB knockout mice with HFD were markedly increased, but there was no significant difference in HDL compared with HDF group (Fig. 4A–D). After PA induction, reducing VEGFB also increased the levels of TG, TCHO and LDL and descended HDL (Fig. 4E–H).

### VEGFB gene suppression aggravated the liver injury of mice in NAFLD

**Fig. 5:**
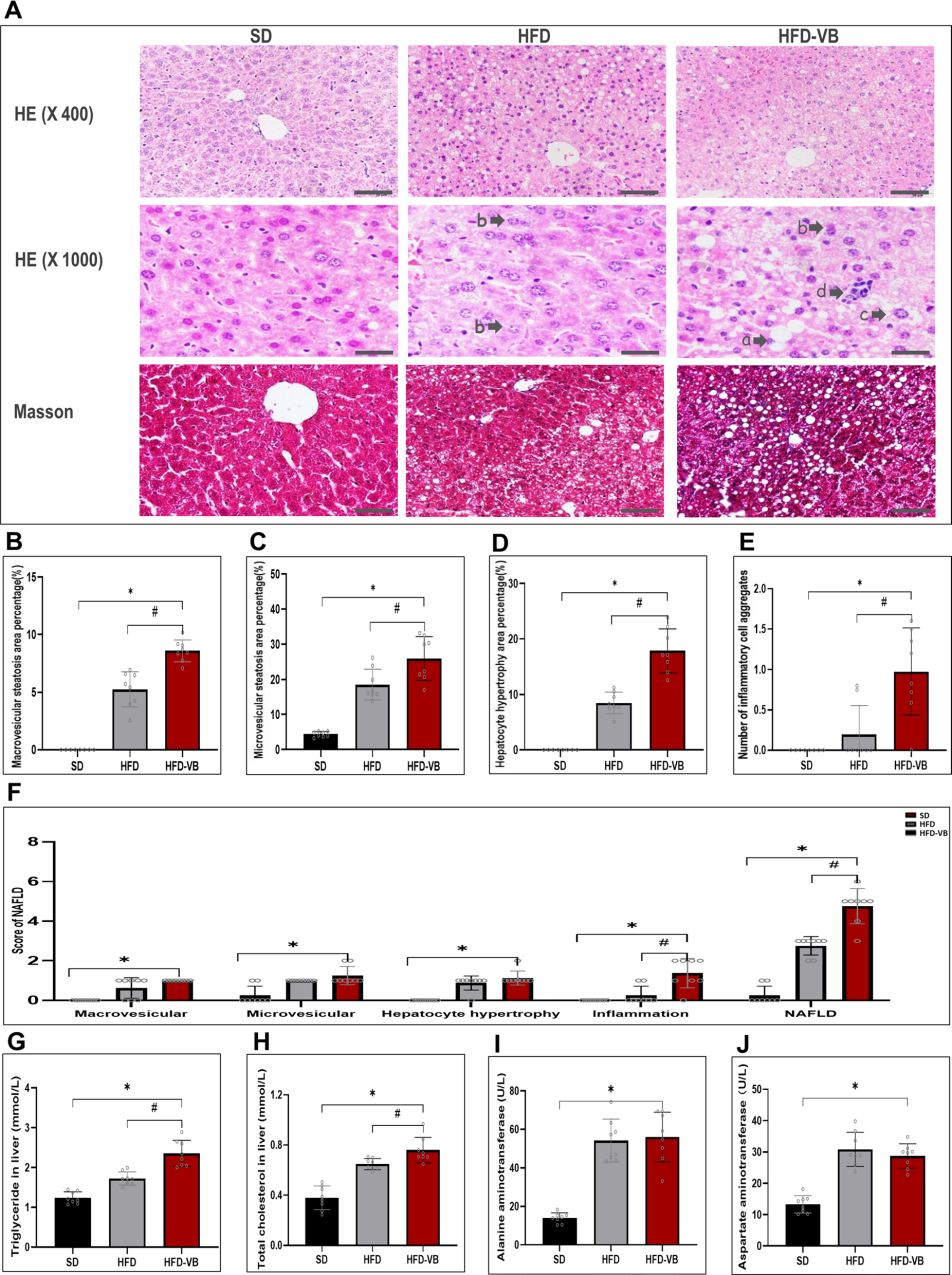
Systemic knockout of VEGFB accelerates liver injury in NAFLD mice. **A** Morphological pictures of mice liver tissue stained by HE and Masson. **a** indicates macrovesicular steatosis, **b** indicates vesicular steatosis, **c** indicates hypertrophic hepatocytes, **d** indicates inflammatory cell aggregates. **B**–**E** Percentage of hepatic bullous steatosis, vesicular steatosis, hypertrophic area of hepatocytes and number of inflammatory cell aggregates in mice liver tissue. **F** Score of hepatic bullous steatosis, vesicular steatosis, hypertrophic area of hepatocytes and inflammatory cell aggregates and total score of NAFLD. **G**, **H** TG and TCHO levels in liver tissue. **I**, **J** AST and ALT levels in mice serum. N = 8 cases per group. *indicates vs. SD, p < 0.05; # indicates vs. HFD, p < 0.05

After mice liver tissue section with HE and Masson staining, the structure of hepatocytes and the degree of fibrosis were observed morphologically. After HFD, the liver injury of NAFLD appeared. The hepatocytes edema and ballooning degeneration in VEGFB knockout mice were more distinct, it could be observed that distinct bullous steatosis, necrosis of single or scattered hepatocytes as well as infiltration of lymphocytes and macrophages around hepatocytes. A small amount of scattered fibrous tissue can be seen in the hepatic lobules of VEGFB knockout mice, and there is no bridging fibrotic change around the lobules and perisinusoidal space (Fig. 5A). We selected bullous steatosis, microtubular steatosis, hepatocyte hypertrophy and inflammatory cell aggregation as the key indicators of NAFLD score. The scoring results revealed that compared with the SD and the HFD groups, VEGFB knockout mice hepatocyte bullae and microtubular showed observable steatosis changes, and hypertrophic hepatocytes and inflammatory cell aggregation also increased dramatically. We evaluated the liver injury of mice according to the NAFLD scoring standard. Compared with the SD group, scores of all items in NAFLD of VEGFB knockout mice were increased, while there were significant differences in inflammatory cell aggregation score and total NAFLD score compared with the HFD group (Fig. 5B–F). By detecting the contents of TG and TCHO in liver tissue, we found that the lipid deposition in liver tissue of VEGFB knockout mice was markedly high (Fig. 5G, H). Meanwhile, the alanine transaminase (ALT) and aspartate transaminase (AST) in VEGFB knockout mice were also high, suggesting liver function injury in NAFLD mice (Fig. 5I, J).

### VEGFB gene knockout exacerbated insulin resistance in mice with HFD

**Fig. 6:**
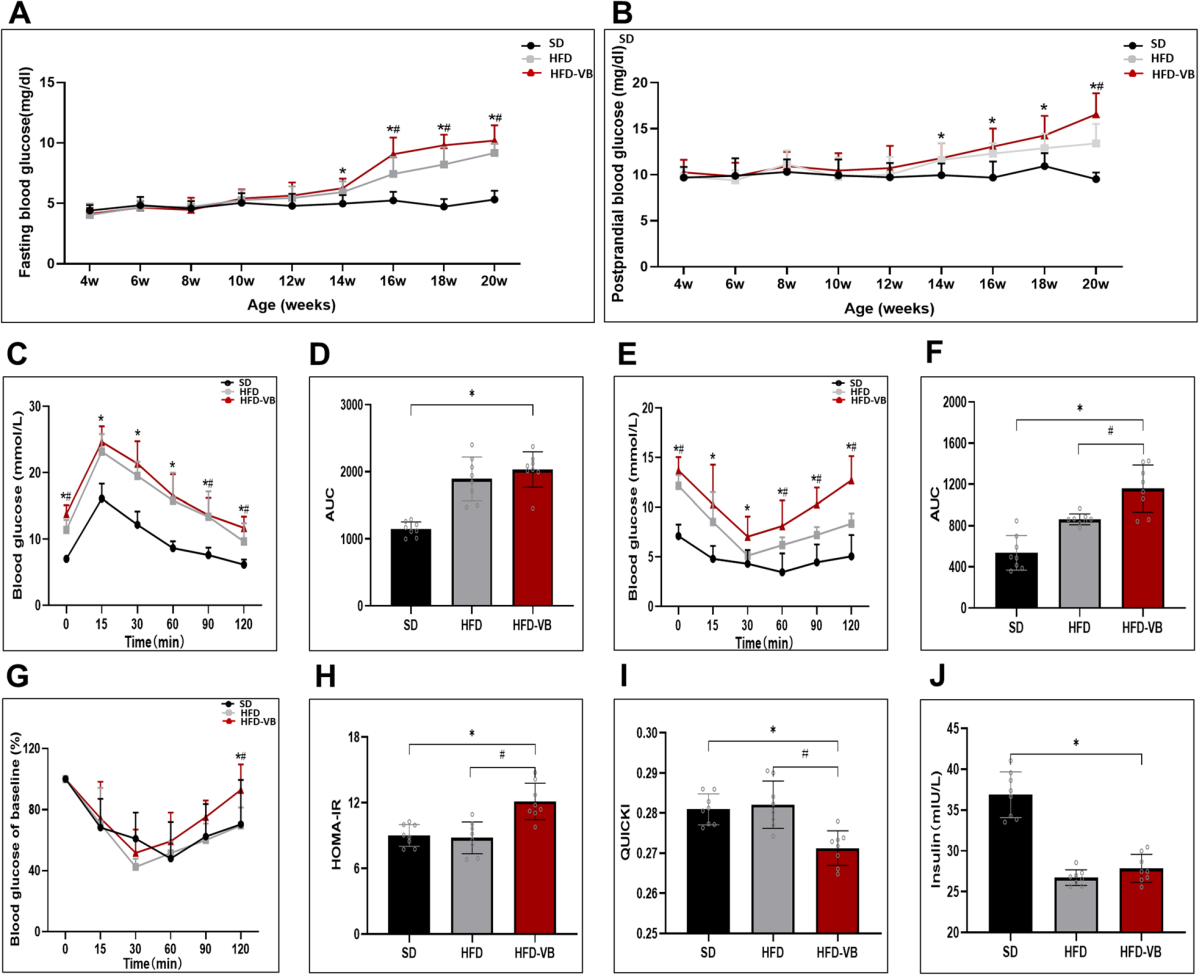
VEGFB gene knockout accelerates insulin resistance in mice. **A**, **B** Curve monitoring of FBG and PBG in 4–20 week-old mice. **C** GTT. **D** Area of GTT under the curve. **E** ITT. **F** Area of ITT under the curve. **G** The relative value of blood glucose decrease. **H** Index of insulin resistance. **I** Insulin sensitivity index. N = 8 cases per group. **J** Serum insulin level after glucose stimulation for 30 min. *indicates vs. SD, p < 0.05; # indicates vs. HFD, p < 0.05

We monitored the indicators of mice, for example, blood glucose and insulin. From the 14th week, the fasting blood glucose and postprandial blood glucose in HFD-VB group were significantly higher than those in SD group. From the 16th week, the fasting blood glucose of HFD-VB group was higher than that of HFD group, while the postprandial blood glucose of HFD-VB group was only higher than that of HFD group at the 20th week (Fig. 6A, B). The glucose tolerance test (GTT) showed that the blood glucose at 0, 90 and 120 min was high in VEGFB knockout group (Fig. 6C). The results of the insulin tolerance test (ITT) showed that the blood glucose of VEGFB knockout mice induced by HFD was continuously high (Fig. 6E, F). Through the ITT test, we calculated the relative value of blood glucose decline, insulin resistance index (HOMA-IR) and Quantitative insulin sensitivity check index (QUICKI). The HOMA-IR increased markedly and the QUICKI descended (Fig. 6G–I). It was found that detecting the secretion of insulin after 30 min of glucose stimulation, the insulin secretion of the HFD and VEGFB knockout mice was insufficient (Fig. 6J).

### VEGFB participates in the regulation of the AMPK signaling pathway through CaMKKβ

**Fig. 7:**
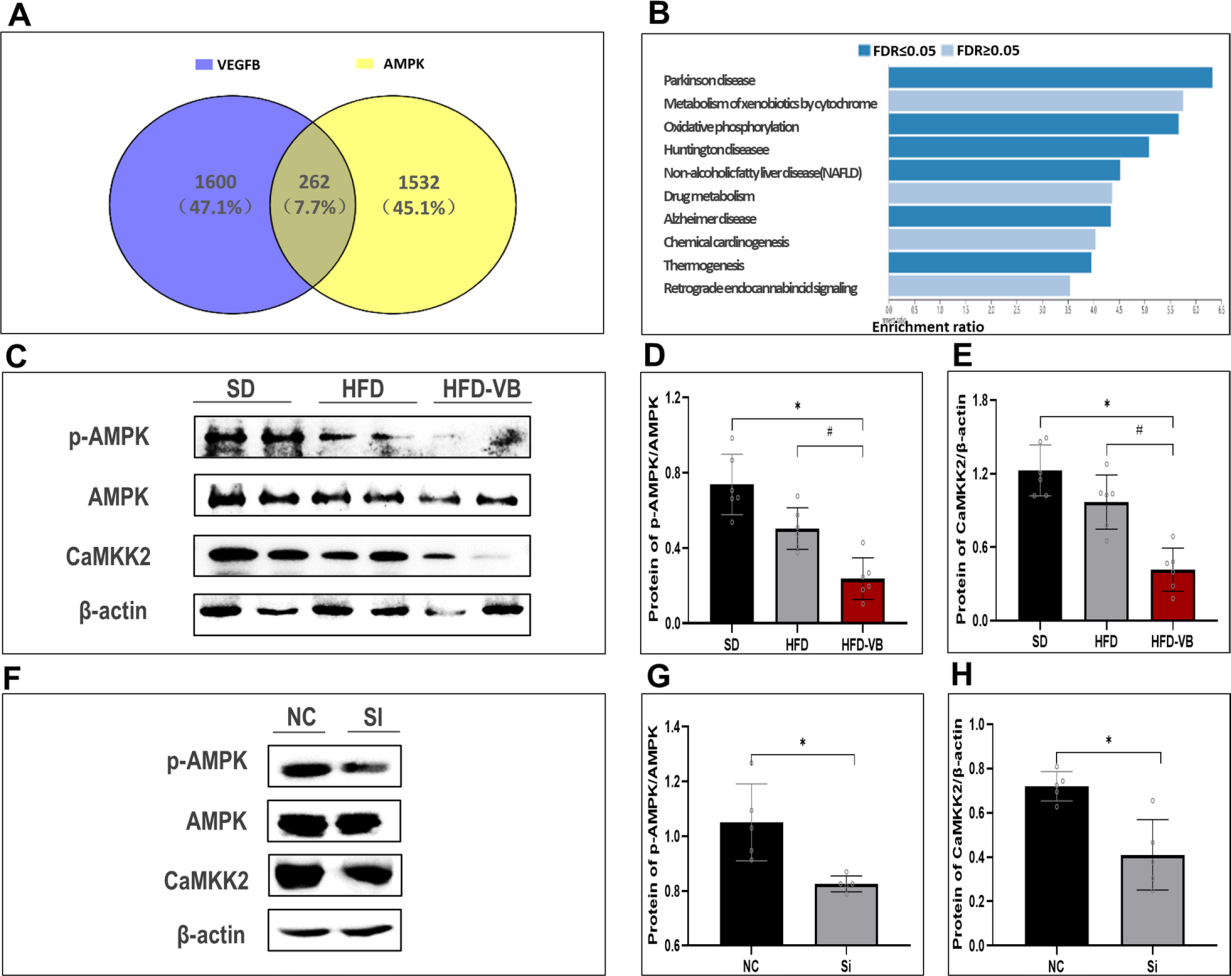
The suppression of VEGFB gene inhibits AMPK phosphorylation level. **A** Venn diagram illustrates the number of loci with gained VEGFB and AMPK binding in liver. **B** Bioinformatics analysis of co-expressed genes of VEGFB and AMPK. **C** p-AMPK, AMPK, CaMKK2 in NAFLD mice model were analyzed by Western blot. **D** Protein expression level of p-AMPK/AMPK in NAFLD mice model. **E** Protein expression level of CaMKK2 in NAFLD mice model. **F** p-AMPK, AMPK, CaMKK2 in NAFLD cell model were analyzed by Western blot. **G** Protein expression level of p-AMPK/AMPK in NAFLD cell model. **H** Protein expression level of CaMKK2 in NAFLD cell model. N = 6 cases per group. **D**, **E** * indicates vs. SD, p < 0.05; ^#^ indicates vs. HFD, p < 0.05. **G**, **H** * indicates vs. NC, p < 0.05

Through Genetwork, we analyzed 2000 genes related to VEGFB and AMPK in normal and high-fat liver. By intersection analysis of these genes, we found that a total of 262 genes were related to them. Through the enrichment analysis of the KEGG pathway, we found that these genes were partially enriched in NAFLD pathway (Fig. 7A, B).

Western blot demonstrated that CaMKKβ and p-AMPK in the liver of VEGFB knockout mice with HFD descended markedly (Fig. 7C–E). The expression levels of CaMKKβ and AMPK phosphorylation in the NAFLD cell model were consistent with the results of VEGFB knockout mice liver tissue (Fig. 7F–H).

### The suppression of VEGFB gene inhibits NAFLD fatty acid oxidation through the AMPK/ACC signaling pathway

**Fig. 8:**
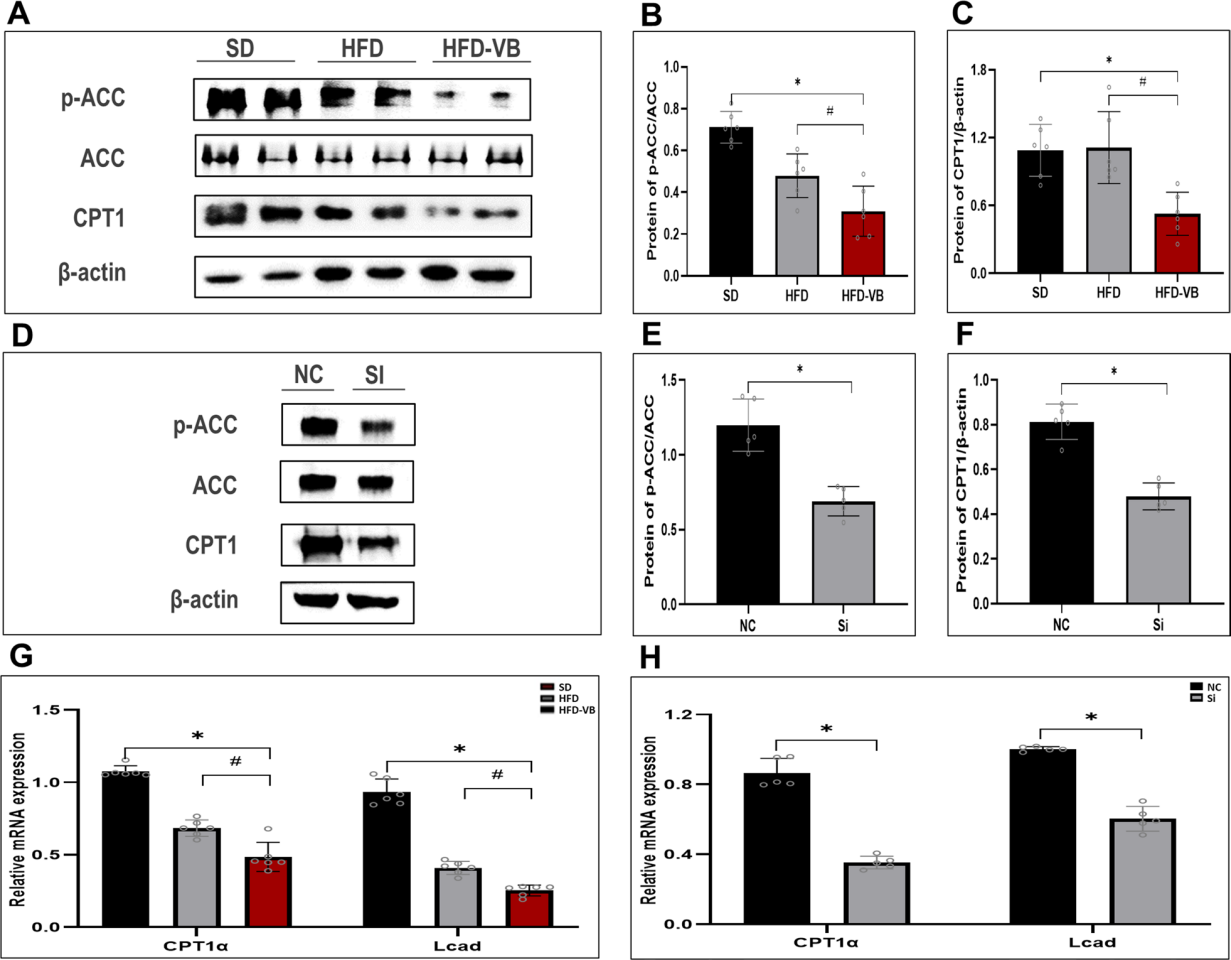
The suppression of VEGFB gene inhibits the AMPK/ACC/CPT1 signal pathway of fatty acid oxidation. **A** Western blot of p-ACC, ACC, CPT1 in NAFLD mice model were analyzed. **B** Protein level of p-ACC/ACC in NAFLD mice model. **C** Protein level of CPT1 in NAFLD mice model. **D** Western blot of p-ACC, ACC and CPT1 protein in NAFLD cell model were analyzed. **E** Protein level of p-ACC/ACC in NAFLD cell model. **F** Protein level of CPT-1 in NAFLD cell model. **G** mRNA level of CPT1α and Lcad in NAFLD mice model. **H** mRNA level of CPT1α and Lcad in NAFLD cell model. N = 6 cases per group. **B**, **C**, **G** *indicates vs. SD, p < 0.05; ^#^ indicates vs. HFD, p < 0.05; **E**, **F**, **H** * indicates vs. NC, p < 0.05

By detecting the key proteins ACC and CPT1 in the fatty acid oxidation pathway, we studied the VEGFB on fatty acid oxidation in NAFLD. The results illustrated that the phosphorylation level of ACC in the liver tissue of VEGFB knockout mice descended distinctly (Fig. 8A, B), and the protein expression of CPT1 also lowered observably (Fig. 8C). The p-ACC and CPT1 in NAFLD cell model were also markedly descended after siRNA transfection for 48 h (Fig. 8D–F). The results of animal and cell models of NAFLD revealed that the expression of CPT1α and Lcad descended markedly after VEGFB gene was suppressed (Fig. 8G, H).

### The suppression of VEGFB gene increases NAFLD lipid synthesis through the AMPK/SREBP1 signaling pathway

**Fig. 9:**
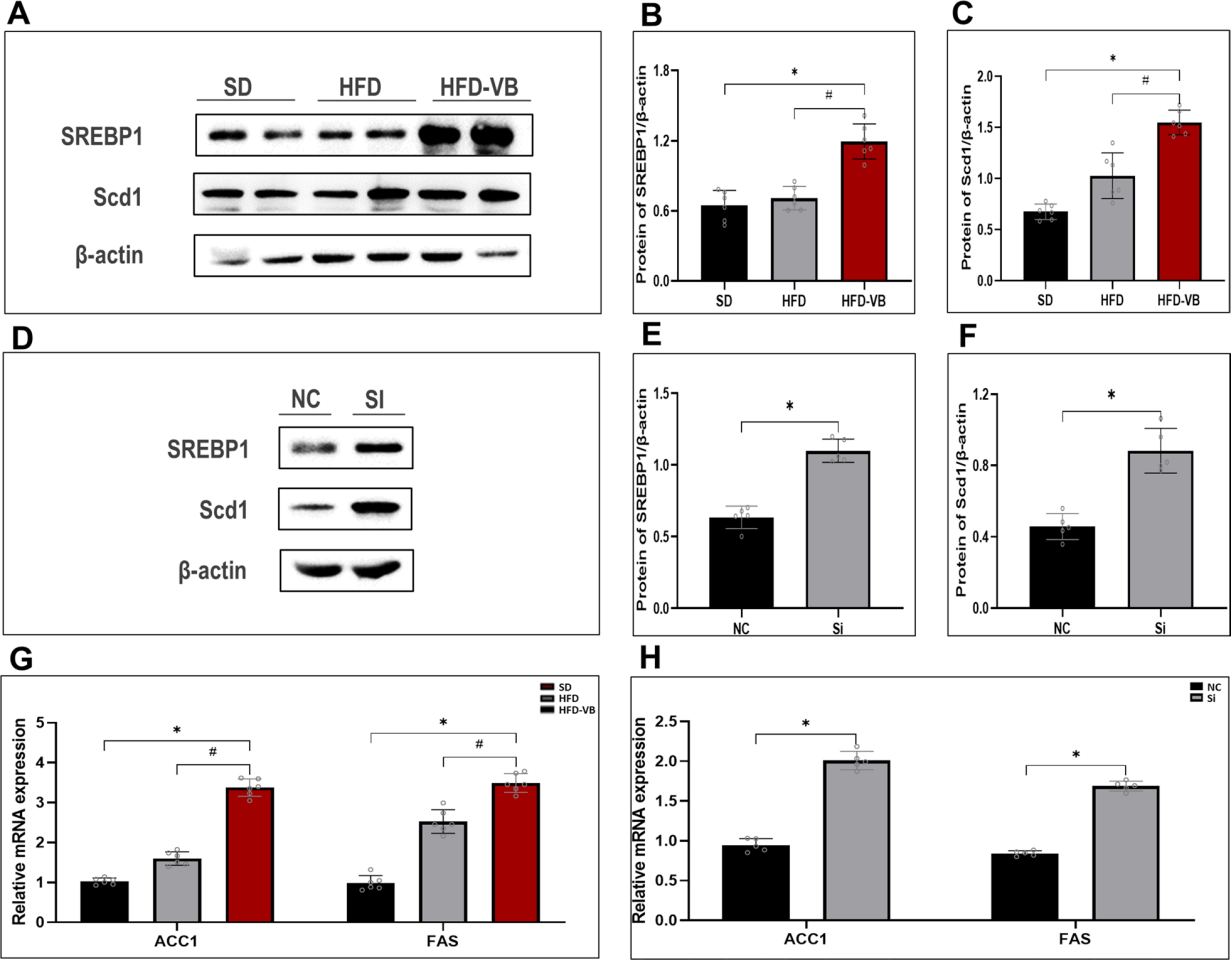
The suppression of VEGFB gene stimulated the SREBP1/Scd1 signal pathway of lipid synthesis. **A** Western blot of SREBP1 and Scd1 in NAFLD mice model were analyzed. **B** Protein level of SREBP1 in NAFLD mice model. **C** Protein level of Scd1 in NAFLD mice model. **D** Western blot of SREBP1 and Scd1 in NAFLD cell model were analyzed. **E** Protein level of SREBP1 in NAFLD cell model. **F** Protein level of Scd1 in NAFLD cell model. **G** mRNA level of ACC1 and FAS in NAFLD mice model. **H** mRNA level of ACC1 and FAS in NAFLD cell model. N = 6 cases per group. **B**, **C**, **G** * indicates vs. SD, p < 0.05; # indicates vs. HFD, p < 0.05; **E**, **F**, **H** * indicates vs. NC, p < 0.05

We also verified the key genes SREBP1 and Scd1 in the lipid synthesis pathway. The protein levels of SREBP1 and Scd1 in liver tissue of VEGFB knockout mice and hepatocytes of NAFLD increased markedly (Fig. 9A–F). We tested ACC1 and FAS, the key genes in lipid synthesis, and the results of animal and cell levels revealed that ACC1 and FAS increased after VEGFB gene was suppressed (Fig. 9G, H).

## Discussion

At present, many studies on NAFLD show that AMPK activation can improve the metabolic pathway of the body, especially has an important regulatory effect on lipid metabolism [20, 21]. Studies have shown that exercise and diet intervention participated in the circulation through AMPK dependent pathway to promote fat phagocytosis of the liver and improve the aging process of the liver, which has become the therapeutic target of NAFLD [22]. Lima et al. found that activating AMPK for phosphorylation can inhibit adipogenesis, promote lipolysis and prevent the development of NAFLD [23].

In 2010, Hagberg et al. first proposed in Nature that VEGFB can control the uptake of fatty acids by endothelial cells through transcriptional regulation of vascular fatty acid transporters [9]. In 2016, Robciuc et al. transduced VEGFB gene into mice and found that VEGFB can inhibit obesity-related inflammatory responses and improve metabolic health [10]. In 2021, Hu et al. found that VEGFB recombinant protein therapy can effectively inhibit the weight gain induced by HFD by activating VEGFR1 [24].

We constructed a NAFLD model of VEGFB gene suppression induced by HFD and PA solution, and clarified the process and mechanism of VEGFB participating in NAFLD fatty acid oxidation and lipid synthesis via acting on the AMPK signaling pathway at the animal and cell level. We found that VEGFB gene suppression accelerated the systemic lipid accumulation in NAFLD mice. After 16 weeks of HFD, it can be seen that VEGFB gene knockout mice have significant increases in body weight, size, fat accumulation and adipocyte morphology. Chen et al. also found that after VEGFB knockout, the obesity phenotype was distinct and the accumulation of white fat increased [25]. We also observed that the blood lipid of C57BL/6 mice with HFD increased, and VEGFB knockout could not undertake the effect of high-fat loaded. The blood lipid level was markedly higher than that of C57BL/6 mice with HFD. Moreover, the TG and TCHO in serum and liver of VEGFB knockout mice with HFD were also markedly higher than those of C57BL/6 mice. After VEGFB gene knockout in the NAFLD cell model, there was also a significant increase in lipid deposition. The results suggested that the conversion speed of free fatty acids increases after VEGFB was knocked out, which can accelerate lipid deposition.

Abnormal lipid deposition in the liver can produce toxic effects, which is easy to cause NAFLD [26]. For example, after hepatocytes are mediated by oxidative stress, lysosomes and mitochondria in cells play an important role in cell damage [27, 28]. When the hepatocytes of NAFLD have foam-like microbubble steatosis, it indicates that there is mitochondrial damage, which will gradually develop into microvesicular steatosis, and a large dominant vacuole will appear in the hepatocytes to replace the nucleus and fill the cells. With the development of steatosis, steatosis hepatocytes appear in the acinar area, leading to more serious liver disease [29]. Studies have shown that VEGFB can affect liver lipids by regulating endothelial fatty acid transport and metabolism [9]. We observed that HFD caused hepatocyte steatosis, and VEGFB gene knockout exacerbated the degree of hepatocyte steatosis. The areas of microbubbles, bullae and mast cells in hepatocytes in the HFD-VEGFB group were markedly increased. The results suggested that VEGFB may be involved in the liver's uptake of lipids from the peripheral circulation and participate in the process of lipid metabolism in vivo through fatty acid transporters [30]. We also observed that there were scattered fibrous tissue and inflammatory cell aggregates in the liver of VEGFB knockout mice. Inflammatory cell aggregates and NAFLD scores were also markedly increased, which accelerated the development of liver injury and NAFLD in mice. Compared with HFD group, there was a significant difference in hepatocyte steatosis in HFD-VB group, but there was no significant difference in the degree of liver fibrosis. There was no significant difference in serum ALT and AST between the two groups, suggesting that VEGFB affected the lipid metabolism of NAFLD mice hepatocytes, and whether VEGFB had an impact on hepatocyte proliferation and apoptosis remains to be further studied.

Hepatic steatosis is epidemiologically close to insulin resistance [31–34]. Obesity and diabetes are usually associated with the development of NAFLD. Studies have shown that liver steatosis occurs first in mice induced by HFD, and then insulin resistance, systemic hyperglycemia and hyperinsulinemia will occur in the liver and adipose tissue [35–37]. Our study showed that in NAFLD mice with VEGFB knockout, blood glucose level increased markedly, glucose tolerance was impaired, insulin resistance was distinct, and insulin sensitivity descended. The reason may be described that in NAFLD mice, insulin resistance is distinct, and the effect of insulin to inhibit lipolysis of adipose tissue is weakened, which leads to increased delivery of free fatty acids to the hepatocytes [38, 39].

Through bioinformatics analysis, we found that CaMKKβ, a key regulatory protein in theAMPK signaling pathway, was associated with VEGFB. CaMKKβ protein in the liver of VEGFB knockout mice with HFD was markedly lower than that of C57BL/6 mice with HFD. Ca^2+^-mediated CaMKKβ activation is a common mechanism of AMPK activation induced by metabolism related hormones [40]. Research showed that the activation of AMPK in LKB1 deficient lung cancer mainly depends on CaMKKβ [41]. AMPK activation in hepatocytes prevents lipid deposition and insulin resistance [42]. AMPK inhibits the fatty acids synthesis and cholesterol by inhibiting the expression of ACC and SREBP1. The overexpression of AMPKα1 in liver of type 2 diabetes rats can also inhibit the expression of adipogenic genes, thereby reducing the content of liver TG and liver steatosis [43].

In 2011, James et al. confirmed that VEGFB could rapidly stimulate the activity of AMPK after incubation with human aortic endothelial cells, and the down-regulation of AMPK could inhibit the response of cells to VEGFB [44]. Our study showed that after VEGFB gene knockout, the p-AMPK protein level and the p-AMPK/AMPK ratio of NAFLD animals and cell models descended markedly. Therefore, we speculated that VEGFB gene suppression can affect the downstream lipid metabolism signaling pathway by inhibiting CaMKKβ activation and reducing AMPK phosphorylation levels.

NAFLD is caused by destructing the balance of liver lipid storage and lipolysis, that is, the disorder of energy balance [45]. AMPK is a sensor of energy changes within cells [46]. When the energy supply changes, AMPK can be activated through the allosteric mechanism to stimulate its kinase activity. Through the phosphorylation of key proteins, AMPK can redirect diverse metabolic directions, including mTOR complex 1 (mTORC1), lipid homeo-metabolism, glycolysis and mitochondrial homeostasis, so as to increase catabolism and decrease anabolism [47–49].

Studies have confirmed that HDL-C is one of the markers of insulin resistance, and insulin resistance can promote the development of NAFLD by inducing accelerated decomposition of triglycerides in adipose tissue and enhanced de novo synthesis of triglycerides in liver [50, 51]. Our study found that VEGFB knockout could accelerate the development of insulin resistance (Fig. 6). In the NAFLD cell model, we detected that the HDL level in Si group was significantly lower than that in NC group (Fig. 4H), suggesting that VEGFB knockdown caused the decrease of HDL-C level, accelerated lipolysis, and increased the level of free fatty acids, resulting in the decrease of insulin sensitivity. There are many factors affecting HDL-C oxidative stress in animals, and the mechanism of the connection between HDL-C and NAFLD has not been fully clarified. In the animal model of NAFLD, we did not observe any significant difference in HDL-C levels between HFD-VB and HFD groups, which may be due to the influence of individual differences and other factors. Whether there is an independent connection between HDL-C and NAFLD remains to be further studied.

In lipid catabolism, AMPK can stimulate the decomposition of macromolecules to produce energy [52]. Free fatty acids depend on the phosphorylation of AMPK and ACC to reduce the expression of malonyl CoA, so as to relieve the inhibition of CPT1 and increase the fatty acids that enter into mitochondria for β-oxidization [53]. Studies have shown that under the influence of many factors, the expression of VEGFB will promote mitochondrial biogenesis and ensure the uptake of fatty acids [54]. We observed that the p-AMPK of systemic VEGFB knockout mice descended, the phosphorylation level of ACC descended, and the downstream CPT1 expression descended accordingly, indicating that VEGFB gene suppression inhibited the AMPK/ACC/CPT1 pathway and enhanced the inhibitory effect of substrate ACC on CPT1, thus affecting fatty acids oxidation in mitochondria.

In anabolism, SREBP1, a key protein downstream of AMPK, is an important regulatory factor in hepatocytes. It controls lipid synthesis in the endoplasmic reticulum and promotes the positive balance of triacylglycerol by inhibiting mitochondrial transporters [55]. Studies have shown that SREBP1 stimulates lipase in insulin resistance conditions, leading to the increase in fat production [56]. The reason for hepatic steatosis is activating the SREBP1/FAS pathway in NAFLD mice [57, 58]. Lee et al. pointed out that inhibition of SREBP1 could delay the development of diet-induced steatosis in obese mice liver [59]. In the adipogenesis pathway, Scd1, downstream of SREBP1, is elevated in patients with metabolic syndrome such as obesity [60].

We observed that the expression of SREBP1 and its downstream Scd1 in mouse liver increased after HFD, which was consistent with previous studies. Compared with HFD, the expression of SREBP1 and Scd1 in VEGFB knockout mouse liver increased more markedly, suggesting that after VEGFB gene suppression, the AMPK/SREBP1/Scd1 pathway will be activated, resulting in lipogenesis and lipid accumulation, and accelerating the formation of NAFLD [61].

**Fig. 10:**
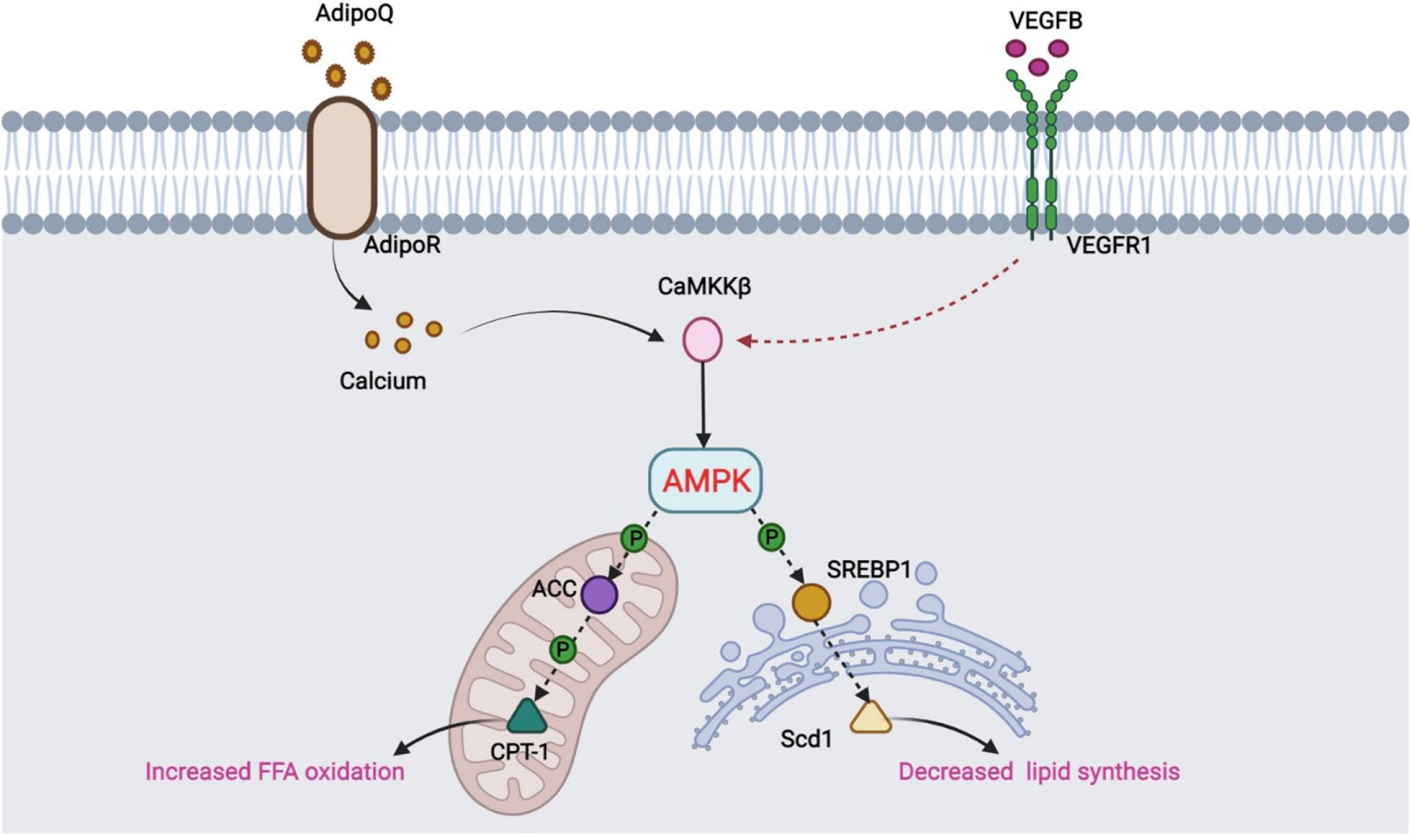
Pattern diagram of VEGFB gene participating in NAFLD through AMPK/ACC and AMPK/SREBP1 signal pathway

Fang et al. found that activation of AMPK can improve oxidative stress, increase fatty acid oxidation and reduce lipid synthesis through the AMPK/ACC and the AMPK/SREBP1 pathways on normal LO2 hepatocytes [14]. We constructed the NAFLD cell model by inducing HepG2 cells with PA to verify the effect of VEGFB gene knockdown on the AMPK/ACC and the AMPK/SREBP1 signaling pathway in hepatocytes. The results were consistent with the animal level, suggesting that VEGFB may participate in the AMPK signaling pathway and lipid metabolism of NAFLD through CaMKKβ. VEGFB may affect fatty acid oxidation of hepatocyte mitochondria through the AMPK/ACC/CPT1 signaling pathway and endoplasmic reticulum lipid production through the AMPK/SREBP1/Scd1 (Fig. 10).

We also analyzed genes closely related to fatty acid metabolism in NAFLD. These target genes play an essential role in lipid metabolism. ACC1 and FAS are used to evaluate de novo synthesis of fatty acid, CPT1α and Lcad are used in mitochondria β oxidztion [62]. We observed that after VEGFB gene suppression, the expression level of CPT1α, the key gene of fatty acid oxidation regulation, and the catalytic enzyme Lcad at the starting site of long-chain fatty acid oxidation descended, but FAS and ACC1, the main lipogenic enzymes working with Scd1, increased markedly.

At present, the commonly used drugs for NAFLD include liver-protecting drugs, insulin sensitizer, lipid-lowering drugs and so on, but the therapeutic effect could not meet the expectation. Nano drug carriers in the treatment of NAFLD have gradually attracted scholars’ interest besides in tumor diagnosis and treatment. Previous studies have shown that in the HepG2 steatosis cell model, liver-targeted Gal-OSL/Res nanocarriers can effectively reduce liver lipid accumulation and reduce insulin resistance by regulating AMPK/SIRT/FAS/SREBP1c signaling pathway. In addition, partially oxidized-reduced nanoparticles also have antioxidant properties, such as zinc oxide nanoparticles [63] and cerium dioxide nanoparticles [64], which can reduce the effect of oxidative stress in the liver, improve the lipid metabolism of NAFLD model mice, and reduce liver lipid accumulation.

In recent years, although the research on nano materials as drug carriers has developed rapidly, nano carrier delivery of NAFLD therapeutic drugs is still in the stage of research in the lab and no clinical trials have been conducted. Our study showed that VEGFB regulates the abnormal lipid metabolism and insulin resistance of NAFLD through AMPK signaling pathway. These findings provided a theoretical and experimental basis for the study of the pathogenesis of NAFLD, and also provided a new idea for the development of a new generation of nano carriers to deliver lipid-lowering drugs and insulin sensitizers for the treatment of NAFLD.

Our study still has some limitations, such as the lack of detection of plasma VEGFB levels in patients with NAFLD, and whether VEGFB has the therapeutic effect of improving or reducing NAFLD liver injury. In the next research, we will collect clinical samples to observe the correlation between the expression of VEGFB and the development of NAFLD, and the further verification of the therapeutic effect of VEGFB up-regulation on NAFLD liver injury through animal and cell experiments. These studies will provide a more sufficient theoretical and experimental basis for the clinical treatment of NAFLD.

## Conclusions

In conclusion, our study proved that VEGFB can participate in lipid metabolism and insulin resistance of NAFLD via the AMPK signaling pathway. In terms of mechanism, VEGFB can affect fatty acid oxidation of hepatocyte mitochondria through the AMPK/ACC/CPT1 signaling pathway, causing changes in the expression of the AMPK/SREBP1/Scd1 key proteins and affecting lipid production in the endoplasmic reticulum (Fig. 10). However, the molecular target of VEGFB in the treatment of NAFLD still needs to be further studied (Additional file 1).

## Supplementary Information

**Additional file 1: Table S1.** Kaplan-Meier curve [65].

## Data Availability

Not applicable.

## References

[1] Friedman SL, Neuschwander-Tetri BA, Rinella M, Sanyal AJ (2018). Mechanisms of NAFLD development and therapeutic strategies. Nat Med.

[2] Fan JG, Kim SU, Wong VW (2017). New trends on obesity and NAFLD in Asia. J Hepatol.

[3] Parikh ND, Marrero WJ, Wang J, Steuer J, Tapper EB, Konerman M (2019). Projected increase in obesity and non-alcoholic-steatohepatitis-related liver transplantation waitlist additions in the United States. Hepatology.

[4] Deprince A, Haas JT, Staels B (2020). Dysregulated lipid metabolism links NAFLD to cardiovascular disease. Mol Metab.

[5] Seebacher F, Zeigerer A, Kory N, Krahmer N (2020). Hepatic lipid droplet homeostasis and fatty liver disease. Semin Cell Dev Biol.

[6] Arruda AP, Pers BM, Parlakgul G, Guney E, Inouye K, Hotamisligil GS (2014). Chronic enrichment of hepatic endoplasmic reticulum-mitochondria contact leads to mitochondrial dysfunction in obesity. Nat Med.

[7] Kim JY, Garcia-Carbonell R, Yamachika S, Zhao P, Dhar D, Loomba R (2018). ER stress drives lipogenesis and steatohepatitis via caspase-2 activation of S1P. Cell.

[8] Olofsson B, Pajusola K, Kaipainen A, von Euler G (1996). Vascular endothelial growth factor B, a novel growth factor for endothelial cells. Proc Natl Acad Sci USA.

[9] Hagberg CE, Falkevall A, Wang X, Larsson E, Huusko J, Nilsson I (2010). Vascular endothelial growth factor B controls endothelial fatty acid uptake. Nature.

[10] Robciuc MR, Kivela R, Williams IM, de Boer JF, van Dijk TH, Elamaa H (2016). VEGFB/VEGFR1-induced expansion of adipose vasculature counteracts obesity and related metabolic complications. Cell Metab.

[11] Zafar MI, Zheng J, Kong W, Ye X, Gou L, Regmi A (2017). Biosci Rep.

[12] Li J, Ding X, Jian T, Lu H, Zhao L, Li J (2020). Four sesquiterpene glycosides from loquat (*Eriobotrya*
*japonica*) leaf ameliorates palmitic acid-induced insulin resistance and lipid accumulation in HepG2 Cells via AMPK signaling pathway. PeerJ.

[13] Garcia D, Hellberg K, Chaix A, Wallace M, Herzig S, Badur MG (2019). Genetic Liver-Specific AMPK Activation Protects against Diet-Induced Obesity and NAFLD. Cell Rep.

[14] Fang K, Wu F, Chen G, Dong H, Li J, Zhao Y (2019). Diosgenin ameliorates palmitic acid-induced lipid accumulation via AMPK/ACC/CPT-1A and SREBP-1c/FAS signaling pathways in LO2 cells. BMC Complement Altern Med.

[15] Ke R, Xu Q, Li C, Luo L, Huang D (2018). Mechanisms of AMPK in the maintenance of ATP balance during energy metabolism. Cell Biol Int.

[16] Zhang W, Li JY, Wei XC, Wang Q, Yang JY, Hou H (2021). Effects of dibutyl phthalate on lipid metabolism in liver and hepatocytes based on PPARalpha/SREBP-1c/FAS/GPAT/AMPK signal pathway. Food Chem Toxicol.

[17] Yi J, Zhu J, Wu J, Thompson CB, Jiang X (2020). Oncogenic activation of PI3K-AKT-mTOR signaling suppresses ferroptosis via SREBP-mediated lipogenesis. Proc Natl Acad Sci USA.

[18] Park M, Baek H, Han JY, Lee HJ (2022). Stevioside enhances the anti-adipogenic effect and beta-oxidation by activating AMPK in 3T3-L1 cells and epididymal adipose tissues of db/db mice. Cells.

[19] Liang W, Menke AL, Driessen A, Koek GH, Lindeman JH, Stoop R (2014). Establishment of a general NAFLD scoring system for rodent models and comparison to human liver pathology. PLoS ONE.

[20] Garcia D, Shaw RJ (2017). AMPK: mechanisms of cellular energy sensing and restoration of metabolic balance. Mol Cell.

[21] Loomba R, Sanyal AJ (2013). The global NAFLD epidemic. Nat Rev Gastroenterol Hepatol.

[22] Gao Y, Zhang W, Zeng LQ, Bai H, Li J, Zhou J (2020). Exercise and dietary intervention ameliorate high-fat diet-induced NAFLD and liver aging by inducing lipophagy. Redox Biol.

[23] Lima RP, Nunes PIG, Viana A, Oliveira FTB, Silva RAC, Alves A (2021). alpha, beta-Amyrin prevents steatosis and insulin resistance in a high-fat diet-induced mouse model of NAFLD via the AMPK-mTORC1-SREBP1 signaling mechanism. Braz J Med Biol Res.

[24] Hu L, Shan Z, Wang F, Gao X, Tong Y (2021). Vascular endothelial growth factor B exerts lipid-lowering effect by activating AMPK via VEGFR1. Life Sci.

[25] Chen Y, Zhao M, Wang C, Wen H, Zhang Y, Lu M (2020). Adipose vascular endothelial growth factor B is a major regulator of energy metabolism. J Endocrinol.

[26] Loomba R, Friedman SL, Shulman GI (2021). Mechanisms and disease consequences of nonalcoholic fatty liver disease. Cell.

[27] Ahmadian E, Eftekhari A, Fard JK, Babaei H, Nayebi AM, Mohammadnejad D (2017). In vitro and in vivo evaluation of the mechanisms of citalopram-induced hepatotoxicity. Arch Pharm Res.

[28] Fard JK, Hamzeiy H, Sattari M, Eftekhari A, Ahmadian E, Eghbal MA (2016). *Triazole*
*rizatriptan* induces liver toxicity through lysosomal/mitochondrial dysfunction. Drug Res.

[29] Cataldo I, Sarcognato S, Sacchi D, Cacciatore M, Baciorri F, Mangia A (2021). Pathology of non-alcoholic fatty liver disease. Pathologica.

[30] Rada P, Gonzalez-Rodriguez A, Garcia-Monzon C, Valverde AM (2020). Understanding lipotoxicity in NAFLD pathogenesis: is CD36 a key driver?. Cell Death Dis.

[31] Musso G, Gambino R, Cassader M, Pagano G (2011). Meta-analysis: natural history of non-alcoholic fatty liver disease (NAFLD) and diagnostic accuracy of non-invasive tests for liver disease severity. Ann Med.

[32] Bae JC, Cho YK, Lee WY, Seo HI, Rhee EJ, Park SE (2010). Impact of nonalcoholic fatty liver disease on insulin resistance in relation to HbA1c levels in nondiabetic subjects. Am J Gastroenterol.

[33] Manchanayake J, Chitturi S, Nolan C, Farrell GC (2011). Postprandial hyperinsulinemia is universal in non-diabetic patients with nonalcoholic fatty liver disease. J Gastroenterol Hepatol.

[34] Dongiovanni P, Stender S, Pietrelli A, Mancina RM, Cespiati A, Petta S (2018). Causal relationship of hepatic fat with liver damage and insulin resistance in nonalcoholic fatty liver. J Intern Med.

[35] Kraegen EW, Clark PW, Jenkins AB (1991). Development of muscle insulin resistance after liver insulin resistance in high-fat-fed rats. Diabetes.

[36] Turner N, Kowalski GM, Leslie SJ, Risis S, Yang C, Lee-Young RS (2013). Distinct patterns of tissue-specific lipid accumulation during the induction of insulin resistance in mice by high-fat feeding. Diabetologia.

[37] Xu H, Barnes GT, Yang Q, Tan G, Yang D, Chou CJ (2003). Chronic inflammation in fat plays a crucial role in the development of obesity-related insulin resistance. J Clin Investig.

[38] Bugianesi E, Gastaldelli A, Vanni E, Gambino R, Cassader M, Baldi S (2005). Insulin resistance in non-diabetic patients with non-alcoholic fatty liver disease: sites and mechanisms. Diabetologia.

[39] Seppala-Lindroos A, Vehkavaara S (2002). Fat accumulation in the liver is associated with defects in insulin suppression of glucose production and serum free fatty acids independent of obesity in normal men. J Clin Endocrinol Metab.

[40] Chauhan AS, Liu X, Jing J, Lee H, Yadav RK, Liu J (2019). STIM2 interacts with AMPK and regulates calcium-induced AMPK activation. FASEB J.

[41] Jin L, Chun J, Pan C, Kumar A, Zhang G, Ha Y (2018). The PLAG1-GDH1 axis promotes anoikis resistance and tumor metastasis through CamKK2-AMPK signaling in LKB1-deficient lung cancer. Mol Cell.

[42] Zang Y, Fan L, Chen J, Huang R, Qin H (2018). Improvement of lipid and glucose metabolism by capsiate in palmitic acid-treated HepG2 Cells via activation of the AMPK/SIRT1 signaling pathway. J Agric Food Chem.

[43] Yan WJ, Wang DB, Ren DQ, Wang LK, Hu ZY, Ma YB (2019). AMPKalpha1 overexpression improves postoperative cognitive dysfunction in aged rats through AMPK-Sirt1 and autophagy signaling. J Cell Biochem.

[44] Feng L, Ren J, Li Y, Yang G, Kang L, Zhang S (2019). Resveratrol protects against isoproterenol induced myocardial infarction in rats through VEGF-B/AMPK/eNOS/NO signalling pathway. Free Radic Res.

[45] Tilg H, Moschen AR, Roden M (2017). NAFLD and diabetes mellitus. Nat Rev Gastroenterol Hepatol.

[46] Li Y, Chen Y (2019). AMPK and autophagy. Adv Exp Med Biol.

[47] Gwinn DM, Shackelford DB, Egan DF, Mihaylova MM, Mery A, Vasquez DS (2008). AMPK phosphorylation of raptor mediates a metabolic checkpoint. Mol Cell.

[48] Leprivier G, Remke M, Rotblat B, Dubuc A, Mateo AR, Kool M (2013). The eEF2 kinase confers resistance to nutrient deprivation by blocking translation elongation. Cell.

[49] Herzig S, Shaw RJ (2018). AMPK: guardian of metabolism and mitochondrial homeostasis. Nat Rev Mol Cell Biol.

[50] Klisic A, Isakovic A, Kocic G, Kavaric N, Jovanovic M, Zvrko E (2018). Relationship between oxidative stress, inflammation and dyslipidemia with fatty liver index in patients with type 2 diabetes mellitus. Exp Clin Endocrinol Diabetes.

[51] Hirschler V, Maccallini G, Sanchez M, Gonzalez C, Molinari C (2015). Association between triglyceride to HDL-C ratio and insulin resistance in indigenous Argentinean children. Pediatr Diabetes.

[52] Hardie DG (2007). AMP-activated/SNF1 protein kinases: conserved guardians of cellular energy. Nat Rev Mol Cell Biol.

[53] McGarry JD, Leatherman GF, Foster DW (1978). Carnitine palmitoyltransferase I. The site of inhibition of hepatic fatty acid oxidation by malonyl-CoA. J Biol Chem.

[54] Mehlem A, Palobo I, Wang X (2016). PGC-1α coordinates mitochondrial respiratory capacity and muscular fatty acid uptake via regulation of VEGF-B. Diabetes.

[55] Raghow R, Yellaturu C, Deng X, Park EA, Elam MB (2008). SREBPs: the crossroads of physiological and pathological lipid homeostasis. Trends Endocrinol Metab.

[56] Shimomura I, Bashmakov Y, Horton JD (1999). Increased levels of nuclear SREBP-1c associated with fatty livers in two mouse models of diabetes mellitus. J Biol Chem.

[57] Shen L, Cui A, Xue Y, Cui Y, Dong X, Gao Y (2014). Hepatic differentiated embryo-chondrocyte-expressed gene 1 (Dec1) inhibits sterol regulatory element-binding protein-1c (Srebp-1c) expression and alleviates fatty liver phenotype. J Biol Chem.

[58] Fang DL, Wan Y, Shen W, Cao J, Sun ZX, Yu HH (2013). Endoplasmic reticulum stress leads to lipid accumulation through upregulation of SREBP-1c in normal hepatic and hepatoma cells. Mol Cell Biochem.

[59] Lee MR, Yang HJ, Park KI, Ma JY (2019). *Lycopus*
*lucidus* Turcz. Ex. Benth. attenuates free fatty acid-induced steatosis in HepG2 cells and non-alcoholic fatty liver disease in high-fat diet-induced obese mice. Phytomedicine.

[60] Zhu X, Bian H, Wang L, Sun X, Xu X, Yan H (2019). Berberine attenuates nonalcoholic hepatic steatosis through the AMPK-SREBP-1c-SCD1 pathway. Free Radic Biol Med.

[61] Quan HY, Kim DY, Kim SJ, Jo HK, Kim GW, Chung SH (2013). Betulinic acid alleviates non-alcoholic fatty liver by inhibiting SREBP1 activity via the AMPK-mTOR-SREBP signaling pathway. Biochem Pharmacol.

[62] Wan J, Jiang L, Lu Q, Ke L, Li X, Tong N (2010). Activation of PPARdelta up-regulates fatty acid oxidation and energy uncoupling genes of mitochondria and reduces palmitate-induced apoptosis in pancreatic beta-cells. Biochem Biophys Res Commun.

[63] Dogra S, Kar AK, Girdhar K, Daniel PV, Chatterjee S, Choubey A (2019). Zinc oxide nanoparticles attenuate hepatic steatosis development in high-fat-diet fed mice through activated AMPK signaling axis. Nanomedicine.

[64] Carvajal S, Perramon M, Oro D, Casals E, Fernandez-Varo G, Casals G (2019). Cerium oxide nanoparticles display antilipogenic effect in rats with non-alcoholic fatty liver disease. Sci Rep.

[65] Ranstam J, Cook JA (2017). Kaplan-Meier curve. Br J Surg.

