# Supplementary figures and images for: Reducing VEGFB accelerates NAFLD and insulin resistance in mice via inhibiting AMPK signaling pathway
Source: J Transl Med. 2022 Jul 30;20:341. doi: 10.1186/s12967-022-03540-2 (PMC9338666; doi:10.1186/s12967-022-03540-2)

**Table S1 Kaplan-Meier curve**

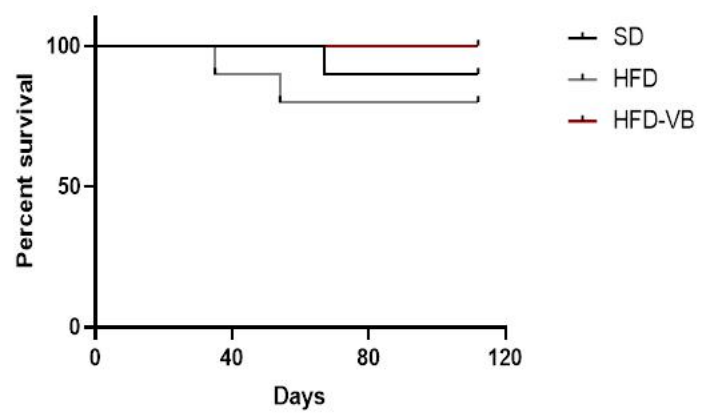

Supplement: Supplementary file 1 — Additional file 1: Table S1. Kaplan-Meier curve [65]. [file 12967_2022_3540_MOESM1_ESM.pdf]
